# Elucidation of the Host Bronchial Lymph Node miRNA Transcriptome Response to Bovine Respiratory Syncytial Virus

**DOI:** 10.3389/fgene.2021.633125

**Published:** 2021-04-22

**Authors:** Dayle Johnston, Bernadette Earley, Matthew S. McCabe, Jaewoo Kim, Jeremy F. Taylor, Ken Lemon, Michael McMenamy, Catherine Duffy, S. Louise Cosby, Sinéad M. Waters

**Affiliations:** ^1^Animal and Bioscience Research Department, Animal and Grassland Research and Innovation Centre, Teagasc, Grange, Ireland; ^2^Division of Animal Sciences, University of Missouri, Columbia, MO, United States; ^3^Veterinary Sciences Division, Agri-Food and Biosciences Institute, Belfast, Northern Ireland

**Keywords:** miRNA, small RNA-Seq, dairy calves, pneumonia, bovine respiratory disease, BRSV challenge

## Abstract

Bovine respiratory disease (BRD) causes substantial morbidity and mortality, affecting cattle of all ages. One of the main causes of BRD is an initial inflammatory response to bovine respiratory syncytial virus (BRSV). MicroRNAs are novel and emerging non-coding small RNAs that regulate many biological processes and are implicated in various inflammatory diseases. The objective of the present study was to elucidate the changes in the bovine bronchial lymph node miRNA transcriptome in response to BRSV following an experimental viral challenge. Holstein-Friesian calves were either administered a challenge dose of BRSV (10^3.5^ TCID_50_/ml × 15 ml) (*n* = 12) or were mock inoculated with sterile phosphate buffered saline (*n* = 6). Daily scoring of clinical signs was performed and calves were euthanized at day 7 post-challenge. Bronchial lymph nodes were collected for subsequent RNA extraction and sequencing (75 bp). Read counts for known miRNAs were generated using the miRDeep2 package using the UMD3.1 reference genome and the bovine mature miRNA sequences from the miRBase database (release 22). EdgeR was used for differential expression analysis and Targetscan was used to identify target genes for the differentially expressed (DE) miRNAs. Target genes were examined for enriched pathways and gene ontologies using Ingenuity Pathway Analysis (Qiagen). Multi-dimensional scaling (MDS) based on miRNA gene expression changes, revealed a clearly defined separation between the BRSV challenged and control calves, although the clinical manifestation of disease was only mild. One hundred and nineteen DE miRNAs (*P* < 0.05, FDR < 0.1, fold change > 1.5) were detected between the BRSV challenged and control calves. The DE miRNAs were predicted to target 465 genes which were previously found to be DE in bronchial lymph node tissue, between these BRSV challenged and control calves. Of the DE predicted target genes, 455 had fold changes that were inverse to the corresponding DE miRNAs. There were eight enriched pathways among the DE predicted target genes with inverse fold changes to their corresponding DE miRNA including: granulocyte and agranulocyte adhesion and diapedesis, interferon signalling and role of pathogen recognition receptors in recognition of bacteria and viruses. Functions predicted to be increased included: T cell response, apoptosis of leukocytes, immune response of cells and stimulation of cells. Pathogen recognition and proliferation of cytotoxic T cells are vital for the recognition of the virus and its subsequent elimination.

## Introduction

The majority of morbidity and mortality reported in calves between 1 and 6 months of age is associated with bovine respiratory disease (BRD) (Taylor et al., 2010; Curtis et al., 2016; Johnston et al., 2016; Murray et al., 2017). The causes of BRD are multifactorial, including infectious viral and bacterial agents, host genetics, farm management, and husbandry practices, environmental stressors including severe weather conditions and the interactions between these factors (Taylor et al., 2010). Viruses are generally the initiators of BRD and the resulting damage they inflict on the respiratory tract predisposes calves to secondary infections through the proliferation and colonisation of bacteria which generally comprise the normal flora of the upper respiratory tract (Johnston et al., 2017).

Bovine respiratory syncytial virus (BRSV) is an enveloped, non-segmented, negative-stranded RNA virus of the *Orthopneumovirus* genus from the family *Pneumoviridae*, and is one of the leading infectious viral causes of BRD (Valarcher and Taylor, 2007; Rima et al., 2017; Sudaryatma et al., 2018). Morbidity resulting from BRSV infections ranges from 60 to 80%, and mortality has been reported to reach 20% during disease outbreaks (Valarcher and Taylor, 2007). BRSV can manifest as sub-clinical or can induce severe clinical signs including coughing, nasal discharge, pyrexia, anorexia and increased respiratory rates (Valarcher and Taylor, 2007). Infection with BRSV leads to the initiation of the inflammatory cytokine response, with increases in several cytokines including IFNγ, IL-12β, IL-6, TNF, IL-18, CXCL8, CCL3, CCL5, CCL2, IFNα1, and IFNβ1, and the subsequent influx of leukocytes, predominantly neutrophils, produces inflammation and lung pathology (Valarcher and Taylor, 2007; Sudaryatma et al., 2018). BRSV is capable of interfering with the host’s anti-viral interferon based response and inducing immunomodulation by shifting the adaptive immune response towards a Th2 dominated response, rather than an effective cytotoxic cell mediated response, which enables establishment and maintenance of the virus (Gershwin, 2007; Valarcher and Taylor, 2007).

Although susceptibility to BRD is moderately heritable (Neibergs et al., 2014), there is limited literature describing the molecular level immune response of the bovine to infection with pathogenic agents of the bovine respiratory disease complex (BRDC), including BRSV. Two RNA-Seq studies of crossbred Angus-Hereford beef calves, conducted in the United States, examined the differentially expressed (DE) genes and pathways in bronchial lymph node (Tizioto et al., 2015) and in multiple lymphoid and lung tissues (Behura et al., 2017) following experimental challenge with single pathogens of the BRDC. Our group has reported 934 genes to be DE in the bronchial lymph node of Irish Holstein-Friesian calves in response to a BRSV challenge (Johnston et al., 2019). However, no studies have been performed to date to elucidate the micro (mi) RNA transcriptional response to an experimental BRSV challenge in calves. Micro RNAs are short, single-stranded, endogenous, non-coding RNA molecules (21–25 nucleotides in length) which are involved in the regulation of gene expression as they trigger the degradation or repress the translation of their target messenger (m) RNAs by directly binding to their 3′ untranslated regions (UTR) (Wahid et al., 2010). Tissue specific miRNA profiles can be altered following infection by various viral and bacterial agents (Lawless et al., 2014; Luoreng et al., 2018). The objective of this study was to elucidate the miRNAs and their target genes involved in the bovine bronchial lymph node transcriptome response to an experimental viral challenge with BRSV in artificially-reared dairy bull calves. These DE miRNAs, and in particular, their target genes which were found to be DE in our previous study (Johnston et al., 2019), may contain variants which influence resistance to BRSV.

## Materials and Methods

### Animal Model

The animal model has previously been described (Johnston et al., 2019). Briefly, Holstein-Friesian bull calves with low BRSV maternally derived antibodies and a negative BRSV PCR result (mean age 143 ± 14 days, mean weight 155 ± 14 kg) were either challenged with 10^3.5^ TCID_50_/ml × 15 ml inoculum of BRSV strain SVA 274/9220 (Graham et al., 1999) (*n* = 12; BRSV challenged) or were mock challenged with sterile phosphate buffered saline (PBS) (*n* = 6; control), by aerosol inhalation, at the Agri-Food Biosciences Institute (AFBI), Stormont, Northern Ireland. Clinical signs including nasal discharge, ocular discharge, general appearance, coughing, respiratory rate and character, size of mandibular lymph nodes, presence or absence of mouth breathing or an expiratory grunt, dyspnoea, and rectal temperature, were recorded daily from the day of challenge until euthanasia, and subsequently scored by a veterinarian (blinded to the calves’ BRSV challenged or control treatment status), using a previously described clinical scoring system (Johnston et al., 2019). Calves were euthanized by captive bolt 7 days post challenge. The lungs were examined and scored for percentages of lesions on the total lung area and on component lung parts, using an AFBI standardised lung scoring system, by a trained veterinarian, as described in Johnston et al. (2019).

Bleach, 75% ethanol and RNaseZap were used to disinfect and remove contaminant RNA/DNA from the work surfaces and implements before tissue collection for each animal. Bronchial lymph node tissues were collected and immediately flash frozen in liquid nitrogen, placed on dry ice and subsequently stored in an −80°C freezer.

### RNA Extraction

The Qiagen RNeasy Plus Universal Mini Kit (Qiagen Ltd., Manchester, United Kingdom), was used according to the manufacturer’s instructions (including Appendix C of the manufacturer’s protocol), for extraction of total RNA (including miRNAs). The quantity and quality of the extracted RNA was determined by measuring the absorbance at 260 nm with a Nanodrop spectrophotometer (NanoDrop technologies; Wilmington, DE, United States) and by using the RNA 6000 Nano LabChip kit (Agilent Technologies Ireland Ltd., Dublin, Ireland) with the Agilent 2100 Bioanalyser (Agilent Technologies Ireland Ltd., Dublin, Ireland). The mean RNA Integrity Number of the samples was 8.6 ± 0.31.

### Library Preparation and Sequencing

Extracted RNA was shipped frozen at −80°C on dry ice to the University of Missouri’s DNA Core Facility for miRNA sequencing library preparation using the TruSeq Small RNA Library Prep Kit (Illumina, San Diego, CA, United States), according to manufacturer’s instructions, and high-throughput sequencing (75 bp) was performed on an Illumina NextSeq 500.

### Bioinformatics and Differential miRNA Expression Analysis

Sequence reads (FASTQ format) were assessed for quality using FastQC (version 0.11.7)^1^. The 3′ ends of the sequence reads were trimmed to remove bases with quality scores less than 20 and (because of artefacts of the NextSeq technology) artificial poly-Gs using cutadapt version 1.18 (Martin, 2011). Reads which contained fewer than 15 bases, greater than 30 bases, or ambiguous nucleotides (N’s) were discarded. Contaminant short RNA sequences including transfer RNAs, ribosomal RNAs, small nucleolar RNAs, and small nuclear RNAs^2^ were removed using Bowtie version 1.2.2 (Langmead et al., 2009). The cleaned sequence reads were analysed for quality metrics using FastQC (version 0.11.7) and all reads passed the basic quality statistics.

Read counts for known miRNAs were generated using the miRDeep2 package (version 2.00.8) (Friedlander et al., 2012) using the UMD3.1 bovine reference genome and the bovine mature and precursor miRNA sequences from the miRBase database (release 22) (Griffiths-Jones et al., 2008). Sequence reads were initially pre-processed (parsed to fasta format, sequences with non-canonical letters discarded, any possible remaining 3′ adapters clipped, short reads discarded), collapsed into clusters and aligned with Bowtie (Langmead et al., 2009) to the indexed UMD3.1 reference genome, using the mapper module (mapper.pl) with default parameters. Subsequently, the miRDeep2 module (miRDeep2.pl) (with default parameters) was used to quantify bovine miRNAs, with *Bos taurus* defined as the species of interest, and with the collapsed reads vs. reference genome alignments, bovine mature miRNA sequences, bovine precursor miRNA sequences (including the hairpin structures) and the human mature miRNA sequences, as input files.

Differential gene expression was determined using the R [R version 3.5.2 (2018-12-20)] Bioconductor package EdgeR (version 3.24.3) (Robinson et al., 2010). This package accounts for both biological and technical variation and models the data using a negative binomial distribution. Any genes with less than one count per million reads in at least six of the samples were removed from the analysis as lowly expressed genes. The trimmed mean of M-values normalisation method (Robinson and Oshlack, 2010) was employed to normalise data across libraries. Dispersion was estimated using both the quantile-adjusted conditional maximum likelihood (qCML) common dispersion and the qCML tagwise dispersion. Exact tests were used for the detection of DE miRNAs between the BRSV challenged and control calves. MiRNAs with a Benjamini–Hochberg false discovery rate (FDR) < 0.1 and a fold change of ≥1.5 were considered DE.

### Target Gene Prediction

Target genes for the DE miRNAs were predicted using the Targetscan 7.0 Perl scripts (Agarwal et al., 2015) downloaded from http://www.targetscan.org/cgi-bin/targetscan/data_download.vert72.cgi. Targetscan_70.pl was used to predict both conserved and non-conserved miRNA target sites using a file containing all known gene transcripts 3′ UTR sequence alignments and the miRNA family information file, as input files. Targetscan_70_BL_bins.pl and targetscan_70_BL_PCT.pl were used to calculate branch lengths and the probability of conservation of target sites. Finally, targetscan_70_context_scores.pl and RNAplfold from ViennaRNA version 2.1.13 were used to calculate the miRNA target genes’ context + + scores.

### Pathway and Functional Enrichment Analysis

The predicted target genes of the DE miRNAs, which were expressed in the bronchial lymph node tissues of the calves had a context ++ percentile rank of 99 and were used for pathway and functional enrichment. Additionally, the predicted target genes for the DE miRNAs which were observed to be DE in the bronchial lymph nodes between the BRSV challenged and control calves with an FDR of <0.1 and a fold change of >2 in the previous study (Johnston et al., 2019) and which had an inverse fold change to that of their miRNA target, were examined for enriched pathways, cellular, and molecular functions and predicted upstream regulators using the Ingenuity Pathway Analysis (IPA) (QIAGEN Inc.^3^), according to the manufacturer’s instructions (Krämer et al., 2014). Identification of over-represented pathways and over-represented molecular and cellular functions was accomplished using Fisher’s exact test, with Benjamini–Hochberg multiple testing correction. The regulation *Z*-score algorithm within IPA, which predicts increases or decreases in functions based on the directional differential expression of genes and expectations derived from the literature, was utilised to predict differences in the over-represented cellular and molecular functions. Cellular and molecular functions with a regulation *Z*-score value of ≥2.0 were predicted to be significantly increased and cellular and molecular functions with a regulation *Z*-score value of ≤−2.0 were predicted to be significantly decreased by the IPA software.

## Results

### Clinical Scores and Lung Pathology

Clinical respiratory sign scoring and lung pathology assessments were performed and results were previously reported in detail in Johnston et al. (2019). Briefly, there were no significant differences in clinical scores between the BRSV challenged and the control calves at any of the time-points (*P* > 0.05), analysed with a repeated measures mixed model procedure in SAS v 9.4. Furthermore, a mixed model ANOVA analysis also performed using SAS v 9.4 indicated that lung scores did not differ between BRSV challenged and control calves (*P* > 0.05). However, the probability of having a lesioned lung was greater for the BRSV challenged than the control calves (*P* = 0.04), determined using a Fisher’s exact test in SAS v 9.4. Lesions were only found on the lungs of one of the control calves, whereas nine of the 12 BRSV challenged calves presented with lung lesions.

### Sequence Alignment

An average of 5,002,047 sequence reads per library was received in FASTQ format. Approximately 2,525,780 reads remained, on average; following trimming with cutadapt and an average of 2,170,434 reads were retained following filtering of reads containing contaminant short RNA species. Following MirDeep2 pre-processing, an average of 2,093,463 sequence reads remained for mapping (Supplementary Table 1). Eighty-eight percent of reads were mapped to the UMD3.1 bovine reference genome (Supplementary Table 1).

### Differential MiRNA Expression

**FIGURE 1:**
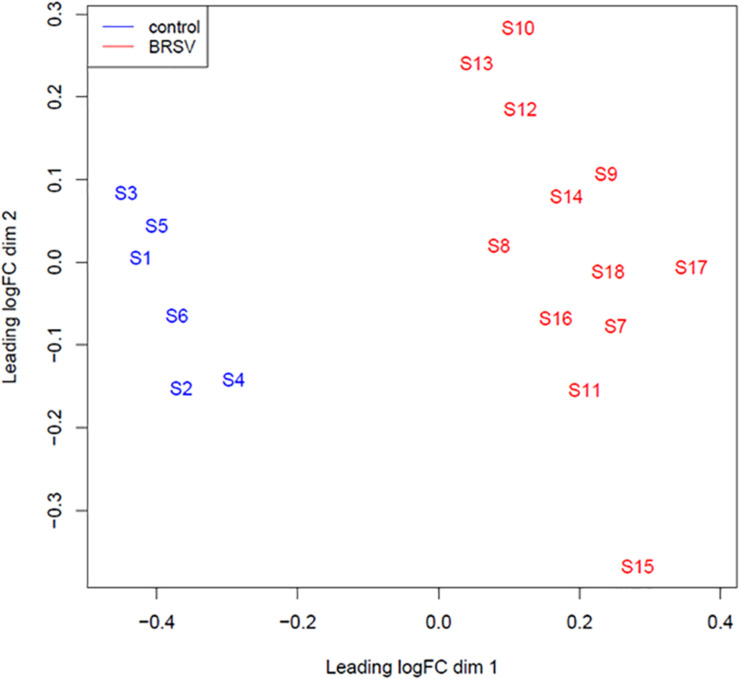
An MDS plot generated in EdgeR illustrating the similarity of the samples based on the Log_2_ miRNA gene expression covariance matrix among individuals. Samples from BRSV challenged calves are coloured in red and samples from control calves are coloured in blue. The numbers (1–18) refer to the calf identifier and the letter S in front of the numbers refers to the word “sample.”

A multi-dimensional scaling (MDS) plot was generated in EdgeR which plotted samples on a two dimensional space based on global miRNA expression, using the first two principal components. A clearly defined separation between the BRSV challenged and the control calves was visible in the MDS plot (Figure 1). There were 119 DE miRNAs (*p* < 0.05, FDR < 0.1, fold change > 1.5) between the BRSV challenged and control calves (Supplementary Table 2). Sixty-seven miRNAs were up-regulated and 52 were down-regulated in the BRSV challenged calves relative to the control calves.

### Target Genes and Functional Annotation of Target Genes

Targetscan predicted that 6,892 of the 13,909 genes which were expressed in the bronchial lymph nodes of these calves (Johnston et al., 2019) were targeted by the 119 DE miRNAs. Of these target mRNA genes, 3,410 had a weighed context + + score percentile of 99% (Supplementary Table 3). The predicted target genes with a weighed context + + score percentile of 99% of the top ten most significant DE miRNAs are presented in Table 1. The predicted target genes with a weighed context + + score percentile of 99% were subsequently analysed for pathway and functional enrichment within IPA. There were 120 enriched pathways among the predicted target genes in the 99th percentile rank of weighted context + + score (FRD < 0.1) (Supplementary Table 4). The most significant of the enriched pathways were associated with IL-8 signalling, cell regulation, apoptosis and cell proliferation (Table 2). There were 500 enriched diseases and functions (FDR < 0.1), and these were related to cell death and survival, inflammation and differentiation (Supplementary Table 5).

**TABLE 1:** The predicted target genes with a weighed context ++ score percentile of 99% of the top ten most significant differentially expressed miRNAs.

**miRNA**	***P*-value**	**FDR**	**Fold change**	**Gene targets**
bta-miR-10164-3p	1.38E-58	6.63E-56	7.75	*SFRP1, MRPL51, CD302, CHIC2, DARS1, PGPEP1, SDC2, FAM241A, RTKN2, EFCAB11, ACER2, SLC25A26, MYOZ1, LPGAT1, PFDN4, SH3BGRL, CAMK2N1, CENPP, KLF9, PPP2R2A, UBTD2, PERP, SYPL1, VPS29, ISCA2, ETFA, ACYP2*
bta-miR-21-3p	1.08E-50	2.60E-48	3.65	*TIMM21, ECT2, ACVR1B, SPRING1, FGF2, TSPAN3, SLC7A11, LSM11, TGFA, ZNF22, NDRG2, TRUB1, PCLAF, SDC2, MPLKIP, FBXO22, ARHGAP12, LYPLA1, C10H14orf119, PAK2, CISD1, HOMER1, NR4A2, RTN3, TUBD1, SKP1, PPTC7, ZBTB14, ATP2B1, PDLIM5, RASSF9, ZBTB41, LMO4, FBXO36, RAB14, TMEM170B, BCL7C, RDH11, PDE1A, ATP13A3, ETF1, MGAT4D, SNAP91, BCL7A, NUDT3*
bta-miR-107	2.11E-27	3.39E-25	2.04	*ST13, ATP5F1E, CAB39, RNF38, CBLN3, TMEM47, GPR15, HPGDS, EIF5A2, BIRC5, NUDCD2, EMB, PLEKHF2, SLC25A5, ZNRF2, SUSD6, DICER1, ACVR2B, TMEM170A, PDLIM5, PPP1R15B, ARMT1, STX7, GOPC, AFG1L, SMNDC1, UBE2A, RRAGC, NIPSNAP3A, NTRK2, ANGPTL7, C8H9orf40, NDUFB6, NLN, EID2, ZHX1, PKIA, STXBP6, PDE7A, CPEB3, SMIM13, LRRC34, SEPTIN5, USF3, LAMTOR3, DDAH1, DNAJC3*
bta-miR-11971	8.00E-25	9.62E-23	3.18	*UBR7, TOMM22, XBP1, CCL22, VPS18, YAE1, NDUFS1, ATP5F1E, CAPNS1, TSPAN3, ESCO1, POLR3K, SLC25A34, ELP6, NUDT21, MSL2, RFFL, OPA3, NFAM1, SOCS3, EFNA5, AP3S2, CMTR2, TMEM167B, SYDE2, SLC20A2, DYNLRB1, EMP2, SPOUT1, TMEM183A, RABIF, HECA, RNGTT, UBL4A, SLC30A7, TACR2, ROMO1, SLC31A1, TBXA2R, SMIM5, EXT1, C13H20orf96, PSMB9, PKIA, SNX20, BLOC1S3, C28H10orf105, PCP4L1, KLHL3, BCL7A*
bta-miR-15a	8.24E-23	7.93E-21	1.90	*CCND1, ASF1A, GPR63, NEK9, YIPF4, NAA50, PRKAB2, RNF144B, RNF138, ARL1, TTC19, SMAD7, MOB3B, N4BP1, FGF2, MGAT4A, PIP4P2, NMNAT2, TRUB1, SPRED1, TRIM35, TMEM192, SULT1B1, CDK5R1, IL20RA, PLAG1, FIBIN, PAGR1, ZNF704, RFLNB, PRMT3, SLC20A2, STX7, GNAI3, DR1, MIGA1, PLPP3, SLC35G1, PPP1R11, KATNAL1, FGF9, TBX20, TMEM100, EMC1, TMEM167A, RAB30, GSKIP, DYNLL2, MOB4*
bta-miR-30d	9.04E-22	6.21E-20	−1.66	*FKBP14, YIPF4, PCNP, TUBD1, MAP3K7, GNG10, CAMK2N1, SPIN1, MINDY3, FAM126B, NSG1, PLEKHM3, TMEM107, LSM5, TRIQK, BICD1*
bta-miR-141	9.03E-22	6.21E-20	2.64	*KRR1, NUDCD1, NFYB, TMX4, CCNB1, SPRING1, AP5M1, STX2, CHIC2, HPS3, OSBPL11, PGRMC2, LSM1, PLAG1, SOBP, VPS37A, C20H5orf22, YAF2, C9H6orf120, CISD1, HORMAD2, IGF1, BRI3BP, SERPINC1, RIT1, TENT5C, DR1, SAR1A, REEP3, UBE2D1, ZDHHC21, EID2, TIA1, TMEM107, MEGF10, UBE2V2, RBM7, VTI1B*
bta-miR-339a	5.85E-18	3.52E-16	−1.69	*ALKBH1, BRMS1L, EGLN3, FDXACB1, BCAT1, CRISPLD2, ANKIB1, IGFBP4, FSTL1, PGRMC2, SPTSSA, C9H6orf120, EFNA5, ABRACL, DR1, METTL8, C8H9orf64, APTX, C28H10orf105, RWDD1, MGAT4D, DYNLL2*
bta-miR-744	1.05E-17	5.62E-16	−2.34	*TMOD2, MRPL35, SH3BGRL3, PPIC, PRMT9, C25H16orf54, MRTO4, SOX18, MANBAL, PLA2G2D4, SMIM5, CENPP, TNFRSF9, LY86, FAXC, C22H3orf18, ABHD14B, RBPMS2, RAB31*
bta-miR-502b	3.70E-17	1.78E-15	−2.40	*CHODL, NAA35, LYPD1, PAFAH1B2, RTCA, TRAM1, FAM219A, EIF2B2, HDAC2, SERBP1, SPOPL, ADSS2, CDYL2, IL6ST, BUB3, C11H2orf68, RAP2A, VAMP4, GATAD1, ENDOD1, DYNLL2, SLC7A11, SLC17A5, SMIM13, ADPRM*

**TABLE 2:** Top enriched pathways among the predicted target genes in the 99th percentile rank of weighted context ++ score.

**Ingenuity canonical pathways**	**−log_10_ (B-H *p*-value)**
Unfolded protein response	3.83
IL-8 signalling	2.24
Molecular mechanisms of cancer	2.24
Cell cycle regulation by BTG family proteins	2.24
fMLP signalling in neutrophils	2.21
Cardiac hypertrophy signalling	2.21
PI3K/AKT signalling	2.21
Protein ubiquitination pathway	2.21
Apoptosis signalling	2.21
CXCR4 signalling	2.08
ERK5 signalling	2.08
Cyclins and cell cycle regulation	2.08
IGF-1 signalling	2.08
TGF-β signalling	2.08
Hypoxia signalling in the cardiovascular system	2.05
Glioma invasiveness signalling	2.01
Breast cancer regulation by stathmin1	1.98

**FIGURE 2:**
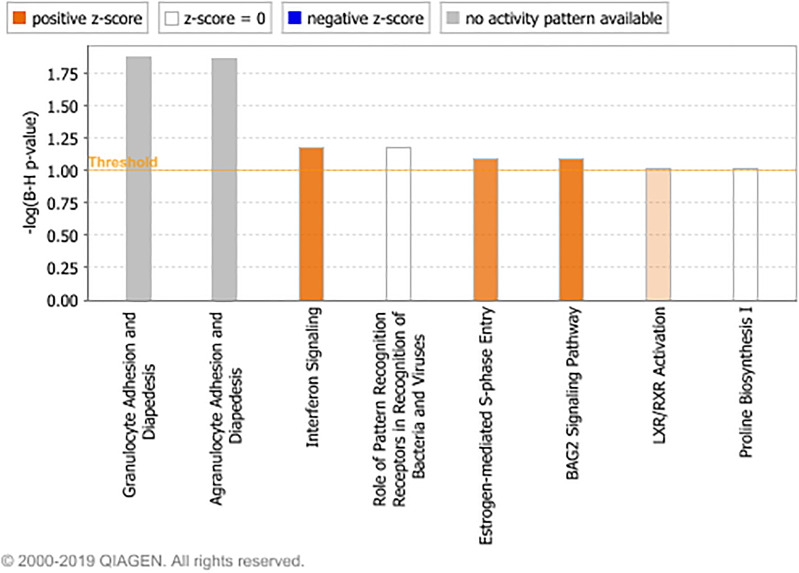
The enriched canonical pathways found in the IPA analysis (*P* < 0.05, FDR < 0.1) among the DE predicted target genes with inverse fold changes to those of the corresponding DE miRNAs. The pathways are shown on the x-axis and the –Log_10_ Benjamini–Hochberg adjusted *p* values are displayed on the y-axis. The threshold is set to one which equals a Benjamini–Hochberg adjusted *p* value of 0.1. Pathways with a positive *z*-score are predicted by IPA to have increased activity and pathways with a negative *z*-score are predicted to have decreased activity.

**FIGURE 3:**
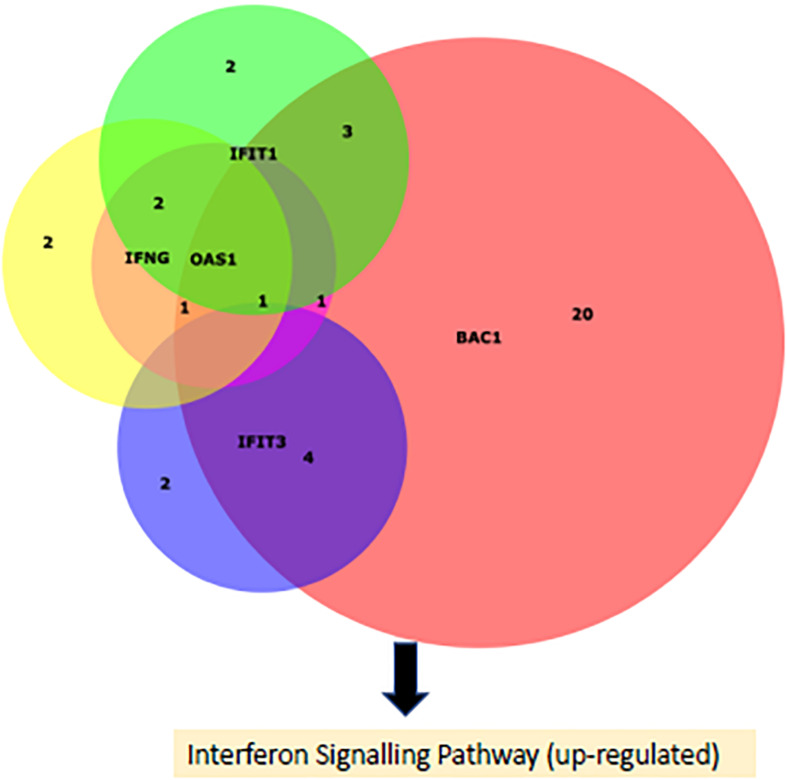
A Venn diagram (Husen et al., 2008) portraying the DE (up-regualted) genes causing the up-regulation of the interferon signalling pathway in the BRSV challenged calves and the number of their targeting DE (down-regulated) miRNAs.

The DE miRNAs were predicted by Targetscan to target 465 genes which were previously found to be DE in bronchial lymph node tissue, between these BRSV challenged and control calves (Johnston et al., 2019). Of the DE predicted target genes, 455 had inverse fold changes to that of the corresponding DE miRNAs (Supplementary Table 6). Ingenuity pathway analysis showed that eight pathways were enriched (FDR < 0.1) among the DE predicted target genes with inverse fold changes to those of the corresponding DE miRNAs including: granulocyte and agranulocyte adhesion and diapedesis, interferon signalling and role of pathogen recognition receptors in recognition of bacteria and viruses (Figure 2 and Supplementary Table 7). Interferon signalling, oestrogen-mediated S-phase entry, BAG2 signalling and LXR/RXR were predicted by IPA to be up-regulated (*Z*-Score ≥ 2) (Figure 2). The up-regulated genes within the interferon signalling pathway and the down-regulated miRNAs targeting these genes are presented in Figure 3 and Table 3. There were 500 enriched diseases and functions (FDR < 0.1), and 21 of these functions were predicted by IPA’s regulation *Z*-score algorithm to be increased (including cell survival, lymphocyte response, T cell response and cell death) (*Z*-Score ≥ 2) while four were predicted to be decreased (including morbidity or mortality) (*Z*-Score ≤ −2) (Supplementary Table 8).

**TABLE 3:** The DE genes within the enriched up-regulated interferon signalling pathway and their DE targeting miRNAs.

**DE Gene**	**Targeting DE miRNA**
*BAK1*	bta-miR-328, bta-miR-504, bta-miR-199b, bta-miR-628, bta-miR-31, bta-miR-148b, bta-miR-6119-3p, bta-miR-95, bta-miR-301a, bta-miR-185, bta-miR-11999, bta-miR-2313-3p, bta-miR-2284w, bta-miR-339a, bta-miR-491, bta-miR-11989, bta-miR-296-3p, bta-miR-2435, bta-miR-1271, bta-miR-183, bta-miR-34b, bta-miR-1249, bta-miR-34c, bta-miR-340, bta-miR-153, bta-miR-2346, bta-miR-744, bta-miR-2415-3p, bta-miR-30b-3p, bta-miR-1343-3p, and bta-miR-7180.
*IFIT1*	bta-miR-148b, bta-miR-2313-3p, bta-miR-129, bta-miR-129-5p, bta-miR-139, bta-miR-183, bta-miR-153, and bta-miR-1296.
*IFIT3*	bta-miR-6119-3p, bta-miR-2284w, bta-miR-183, bta-miR-2346, bta-miR-502b, bta-miR-30b-3p, and bta-miR-11998.
*IFN*γ	bta-miR-328, bta-miR-301a, bta-miR-129, bta-miR-129-5p, bta-miR-183, bta-miR-2284v, and bta-miR-2284d.
*OAS1*	bta-miR-328, bta-miR-129, bta-miR-129-5p, bta-miR-183, and bta-miR-1343-3p.

*The DE genes were all up-regulated in the bronchial lymph node of the BRSV challenged calves and the DE targeting miRNAs were all down-regulated.*

## Discussion

To our knowledge, this is the first study to examine the miRNA transcriptional response in calves to challenge with BRSV. The BRSV experimental challenge induced large changes in miRNA transcription with 119 DE miRNA in bronchial lymph node tissue in BRSV challenged compared to control Holstein-Friesian calves. Micro-RNAs are involved in the post transcriptional regulation of biological responses (Wahid et al., 2010) and in innate and adaptive immune mechanisms (Inui and Martello, 2010; O’Connell et al., 2010; Vegh et al., 2013). They play an important regulatory role in the global translation of genes into proteins as they can reduce levels of mRNA by initiating its degradation or they may prevent its translation (Wahid et al., 2010). They have been implicated in bovine disease conditions, host immune responses to pathogens and environmental challenge. For example, the miRNA profile of dry secretions up to 21 days post dry-off has been characterised in pregnant multi-parous Holstein cows, and changes in miRNA expression over-time were associated with pregnancy, lactation, inflammation and disease (Putz et al., 2019). Additionally, twenty-seven miRNAs were identified to be DE between mammary tissues of Holstein cattle experiencing heat stress and normal conditions (Li et al., 2018).

Several studies have reported changes in the expression of bovine miRNAs due specifically to disease status. Twenty-five miRNAs were DE in milk from Holstein Friesian cows infected with mastitis relative to milk from healthy Holstein Friesian cows (Lai et al., 2020). Furthermore, members of the BRDC have been observed to induce miRNA transcriptional alterations including a change in abundance of two miRNAs in serum, due to a bovine viral diarrhoea virus challenge in colostrum deprived, neonate, Holstein calves (Taxis et al., 2017), and variations in the concentrations of four miRNAs associated with a serum antibody response, which indicated exposure to mycoplasma, in beef cattle (Casas et al., 2016). However, while these studies show the involvement of miRNA in multifactorial aspects of animal health, there is no published literature detailing any alterations in transcription of miRNAs due to a specific viral challenge with BRSV, one of the main viral causes of BRD.

Understanding the changes in abundance of miRNAs during an experimental challenge with BRSV leads to important insights into the host transcriptional and regulatory response to infection. BRSV replicates in ciliated airway epithelial cells and type 2 pneumocytes and induces the expression of host pro-inflammatory cytokines which recruit neutrophils and lymphocytes to the lung and causes bronchiolitis and interstitial pneumonia (Guzman and Taylor, 2015). The observed changes in host miRNA abundance induced by the BRSV challenge demonstrates that miRNAs play a role in the recognition of BRSV and the instigation of the host pro-inflammatory anti-viral state during infection with BRSV. This is evident due to an enrichment of specific pathways and functions among the predicted target genes of the DE miRNA. These include Toll-like receptor signalling, IL-8 signalling, apoptosis signalling, role of MAPK signalling in the pathogenesis of Influenza, JAK/Stat signalling, NF-kB signalling, T cell receptor signalling, chemokine signalling, apoptosis, cell death of lung cells, T cell development, and antiviral response of T lymphocytes.

In the current study, the miRNAs which were DE in response to the BRSV challenge infection targeted 455 genes which were DE in the bronchial lymph node of these calves (Johnston et al., 2019) and which had inverse fold changes to those of the DE miRNA. Since the miRNAs reduce the concentration and/or the level of translation of their target mRNA transcripts, the DE miRNAs whose fold changes are inversely related to the fold changes of their target DE mRNAs show a consistent functional response outcome and therefore are the most relevant target mRNAs of the DE miRNAs. These target genes were involved in the recognition of BRSV by pathogen recognition receptors, granulocyte and agranulocyte adhesion, and diapedesis and interferon signalling. These enriched pathways among the DE predicted target genes of the DE miRNA show that miRNAs are important in the transcriptional response to BRSV infection and that the response is broadly similar to that of the host response to human respiratory syncytial virus (HRSV) which is primarily associated with the activation of interferon gamma associated signalling pathways (Vieira et al., 2019) and recruitment of cytotoxic T cells which are capable of destroying virus infected cells and resolving the infection (Griffiths et al., 2017). Furthermore, the host immune response, including the production of pro-inflammatory cytokines and the influx of neutrophils, natural killer cells and cytotoxic T cells, are responsible for the majority of respiratory tissue pathology, rather than virus replication (Griffiths et al., 2017). However, the virus causes some cytopathology, including in bronchial epithelial cells, through the creation of intracytoplasmic inclusion bodies and large multinucleated syncytial cells (Griffiths et al., 2017).

Calves are natural hosts for BRSV, the BRSV calf challenge infection is also considered to be a model system for HRSV infection in infants (Guzman and Taylor, 2015; Zhang et al., 2017). HRSV is an important cause of respiratory disease mortality in children younger than 5 years of age (Scheltema et al., 2017). Globally, HRSV is responsible for the deaths of approximately 60,000 children aged younger than 5 years, per annum (Battles and McLellan, 2019). BRSV and HRSV use their non-structural proteins to supress their host’s induction of an anti-viral type 1 interferon (IFN) response (Guzman and Taylor, 2015). They accomplish this through interference with IFN signal transduction by decreasing the levels of STAT2 (Ramaswamy et al., 2004; Janssen et al., 2007) and inhibiting the activation and nuclear translocation of IFN regulatory factor 3 (Spann et al., 2005). They also use their G and small hydrophobic proteins to evade the host’s innate and acquired immune responses (Guzman and Taylor, 2015), including interference with Toll like receptor signalling (Moore et al., 2008), promotion of a Th2 primed response (Griffiths et al., 2017), the down-regulation of expression of IFNβ and ISG15 (Moore et al., 2008), induction of SOCS protein expression (Moore et al., 2008; Bakre et al., 2012; Zheng et al., 2015) and inhibition of TNF-α signalling (Fuentes et al., 2007). However, we (Johnston et al., 2019) and others (Janssen et al., 2007) have shown that even though BRSV and HRSV can suppress the type 1 interferon anti-viral host response, the host is still capable of mounting a primarily Th1 skewed, interferon dominated, transcriptional immune response, due to the up-regulation of IFN-γ and of interferon stimulated genes. The present study further emphasises the importance of interferon signalling in the host response to BRSV as interferon signalling was predicted to be up-regulated in response to the BRSV experimental challenge among the DE miRNA target genes. Additionally, it highlights an important role of miRNAs in regulating and augmenting the interferon anti-viral response.

In the current study, 39 miRNAs involved in the regulation of the interferon response to BRSV were discovered. Of these DE miRNAs, several of them targeted multiple up-regulated genes involved in the interferon response. *miR-183* targeted *IFNγ*, *IFIT*, *IFIT3*, *BAK1* while *OAS1, miR-328*, and *miR-129-5p* targeted *IFNγ*, *BAK1*, and *OAS1, mir-129* targeted *IFNγ*, *IFIT1*, and *OAS1, miR-1343-3p* targeted *BAK1* and *OAS1, miR-301a* targeted *BAK1* and *IFNγ*, *miR-2284w* and *miR-30b-3p* targeted *BAK1* and *IFIT3*, and *miR-148b, miR-2313-3p*, and *miR-153* targeted *BAK1* and *IFIT1*. Interestingly, these DE miRNAs were all down-regulated, while all their targets were up-regulated. Therefore, these miRNAs are likely involved in regulating the interferon response and a decrease in expression of these miRNAs is conceivably responsible for the increased expression of these genes (*IFNγ*, *IFIT*, *IFIT3*, *BAK1*, and *OAS1*) involved in the interferon response.

Although there are no published studies to-date detailing the miRNA transcriptional response to BRSV, there are several studies describing changes in miRNA abundances due to HRSV infection, including the up-regulation of the miRNAs; *let-7f*, *miR-24*, *miR-337-3p*, *miR-26b*, and *miR-520a-5p*, and the down-regulation of *miR-198* and *miR-595*, *in vitro*, in a human alveolar epithelial cell line (Bakre et al., 2012). However, the majority of changes in miRNA expression induced by HRSV described in published studies (Leon-Icaza et al., 2019) were not observed in this study. Indeed, several *in vitro* studies have found members of the miR-30 family to be up-regulated in response to HRSV infection (Wu et al., 2020) whereas in this current study, *miR-30b-3p*, *miR-30d*, *miR-30f*, and *miR-30c*, were down-regulated in bovine bronchial lymph node tissue following the *in vivo* experimental challenge with BRSV. A possible reason for the inconsistencies observed between changes in expression of miRNAs due to BRSV in this study and previous HRSV studies, may be that this study examined changes in bronchial lymph node tissue from calves while the other studies were performed using cell lines or cultured primary or dendritic cells, *in vitro* (Bakre et al., 2012; Thornburg et al., 2012; Leon-Icaza et al., 2019). Interestingly, *miR-139* was similarly down-regulated in human bronchial epithelial cell culture due to infection with HRSV (Othumpangat et al., 2012) as in bronchial lymph node tissue due to the BRSV challenge in this present study. Additionally, changes in expression of *miR-30d*, *miR-34b*, and *miR34c*, were consistent between this study and a study which found that these three miRNAs had reduced expression in nasal mucosa from HRSV clinically-infected infant patients compared to healthy controls (Inchley et al., 2015). Furthermore, these three miRNAs were also down-regulated in airway bronchial epithelial cell brushings from asthmatic adult patients (Solberg et al., 2012), which supports their potential importance in respiratory health.

Currently, there are no licenced vaccines for HRSV, despite more than 50 years of research into potential candidate vaccines (Atherton et al., 2019). Furthermore, despite the existence of several vaccines targeting BRSV since the 1970s, there is a lack of evidence supporting their efficacy in reducing disease incidence or morbidity or mortality (Ellis, 2017). Therefore, knowledge of potential miRNA biomarkers of BRSV based on the host response to challenge infections as in the current study may be beneficial for predicting vaccine candidate outcomes. Furthermore, the development of new therapeutic anti-viral treatments targeting miRNAs which stimulate the pro-inflammatory response would be beneficial as damage induced by the host’s own immune response causes the majority of BRSV induced pathologies observed in respiratory tissues (Valarcher and Taylor, 2007).

The miRNAs involved in the bovine bronchial lymph node response to BRSV elucidated in this study could, following further validation, possibly be used as new diagnostic tools for BRSV since the differently abundant miRNAs may act as biomarkers of BRSV disease exposure. This novel form of diagnostic assay would be particularly useful since BRSV can often present as sub-clinical disease, as was evidenced in the present study. However, despite the observation of only a mild clinical response to BRSV infection in the current study, there were large changes in both mRNA and miRNA transcription in the bronchial lymph node of calves responding to the BRSV challenge. Therefore, the DE miRNAs and the DE mRNAs observed in this study and the previous study (Johnston et al., 2019), could potentially be used to develop a novel diagnostic test for BRSV before full symptoms of the disease manifest.

The miRNAs involved in the calves’ bronchial lymph node global transcriptomic regulatory response to BRSV, and their target genes, likely harbour variants which influence susceptibility to BRD. The identification of microRNAs changed in the challenged BRSV calf model will help to elucidate the complex inflammatory response in BRD and should contribute to our understanding of its pathogenesis. The DE miRNA and their target genes could be interrogated further in large scale longitudinal studies where calves acquire BRD either through natural infection or experimental challenge with other BRD causing pathogens. Following validation, variants in these DE miRNAs and their target genes could be included in genomic selection breeding programmes to contribute to the breeding of healthier cattle with improved resistance to BRD. In conclusion, this is the first report on the miRNA expression profile in BRSV challenged calves and may provide a basis for revealing the regulatory mechanism of BRSV infection and the potential role of miRNAs as biomarkers of BRD diagnosis.

## Data Availability Statement

All sequence data produced in this study has been deposited to NCBI GEO repository and are available through the series accession number GSE151033.

## Ethics Statement

The animal study was reviewed and approved by United Kingdom Animals (Scientific Procedures) Act 1986 and with the approval of the Agri-Food and Biosciences Institute Northern Ireland Ethical Review Committee.

## Author Contributions

SW, JT, JK, BE, and SC conceived and designed the experiments. KL, CD, MM, and SC developed and executed the animal challenge model. DJ, KL, CD, MM, JK, SW, BE, and MM performed the experiments. DJ performed the bioinformatics, analysed the data, and wrote the manuscript. All authors reviewed the manuscript. All authorscontributed to the article and approved the submitted version.

## Conflict of Interest

The authors declare that the research was conducted in the absence of any commercial or financial relationships that could be construed as a potential conflict of interest.

## Footnotes

1 http://www.bioinformatics.babraham.ac.uk/projects/fastqc/

2 Downloaded from https://rnacentral.org/

3 https://www.qiagenbioinformatics.com/products/ingenuitypathway-analysis

## Supplementary Material

Supplementary Table 1 Read counts after each processing step and after mapping to the UMD3.1 reference genome and MirBase 22 miRNA sequences.

Supplementary Table 2 DE miRNAs in the bronchial lymph node between BRSV challenged and control calves.

Supplementary Table 3 Target mRNA genes with a weighed context + + score percentile of greater than 99%.

Supplementary Table 4 Enriched pathways among the predicted target genes with a 99% weighted context ++ score rank.

Supplementary Table 5 Enriched diseases and functions among the predicted target genes in the 99th percentile rank of weighted context + + score (FRD < 0.1).

Supplementary Table 6 The predicted target genes which were previously found to be DE in the bronchial lymph node with their corresponding fold changes, *P* values, FDR values and their DE target miRNAs.

Supplementary Table 7 Enriched IPA pathways among the DE predicted target genes with inverse fold changes to that of the DE miRNA.

Supplementary Table 8 Enriched diseases and functions among the DE predicted target genes with inverse fold changes to that of the DE miRNA.

## References

[B1] Agarwal V. Bell G. W. Nam J.-W. Bartel D. P. (2015). Predicting effective microRNA target sites in mammalian mRNAs. *eLife* 4:e05005. 10.7554/eLife.05005 26267216 PMC4532895

[B2] Atherton L. J. Jorquera P. A. Bakre A. A. Tripp R. A. (2019). Determining immune and miRNA biomarkers related to respiratory Syncytial Virus (RSV) vaccine types. *Front. Immunol.* 10:2323. 10.3389/fimmu.2019.02323 31649663 PMC6794384

[B3] Bakre A. Mitchell P. Coleman J. K. Jones L. P. Saavedra G. Teng M. (2012). Respiratory syncytial virus modifies microRNAs regulating host genes that affect virus replication. *J. Gen. Virol.* 93 (Pt 11), 2346–2356. 10.1099/vir.0.044255-0 22894925 PMC3542124

[B4] Battles M. B. McLellan J. S. (2019). Respiratory syncytial virus entry and how to block it. *Nat. Rev. Microbiol.* 17 233–245. 10.1038/s41579-019-0149-x 30723301 PMC7096974

[B5] Behura S. K. Tizioto P. C. Kim J. Grupioni N. V. Seabury C. M. Schnabel R. D. (2017). Tissue tropism in host transcriptional response to members of the bovine respiratory disease complex. *Sci. Rep.* 7:17938. 10.1038/s41598-017-18205-0 29263411 PMC5738336

[B6] Casas E. Cai G. Kuehn L. A. Register K. B. McDaneld T. G. Neill J. D. (2016). Association of MicroRNAs with antibody response to *Mycoplasma bovis* in beef cattle. *PLoS One* 11:e0161651. 10.1371/journal.pone.0161651 27537842 PMC4990326

[B7] Curtis G. C. Argo C. M. Jones D. Grove-White D. H. (2016). Impact of feeding and housing systems on disease incidence in dairy calves. *Vet. Rec.* 179:512. 10.1136/vr.103895 27803374 PMC5299099

[B8] Ellis J. A. (2017). How efficacious are vaccines against bovine respiratory syncytial virus in cattle? *Vet. Microbiol.* 206 59–68. 10.1016/j.vetmic.2016.11.030 28024854

[B9] Friedlander M. R. Mackowiak S. D. Li N. Chen W. Rajewsky N. (2012). miRDeep2 accurately identifies known and hundreds of novel microRNA genes in seven animal clades. *Nucleic Acids Res.* 40 37–52. 10.1093/nar/gkr688 21911355 PMC3245920

[B10] Fuentes S. Tran K. C. Luthra P. Teng M. N. He B. (2007). Function of the respiratory syncytial virus small hydrophobic protein. *J. Virol.* 81 8361–8366. 10.1128/JVI.02717-06 17494063 PMC1951288

[B11] Gershwin L. (2007). Bovine respiratory syncytial virus infection: immunopathogenic mechanisms. *Anim. Health Res. Rev.* 8 207–213. 10.1017/s1466252307001405 18218161

[B12] Graham D. A. Foster J. C. Mawhinney K. A. Elvander M. Adair B. M. Merza M. (1999). Detection of IgM responses to bovine respiratory syncytial virus by indirect ELISA following experimental infection and reinfection of calves: abolition of false positive and false negative results by pre-treatment of sera with protein-G agarose. *Vet. Immunol. Immunopathol.* 71 41–51. 10.1016/s0165-2427(99)00086-0 10522785 PMC7119899

[B13] Griffiths C. Drews S. J. Marchant D. J. (2017). Respiratory syncytial virus: infection, detection, and new options for prevention and treatment. *Clin. Microbiol. Rev.* 30 277–319. 10.1128/cmr.00010-16 27903593 PMC5217795

[B14] Griffiths-Jones S. Saini H. K. van Dongen S. Enright A. J. (2008). miRBase: tools for microRNA genomics. *Nucleic Acids Res.* 36 D154–D158. 10.1093/nar/gkm952 17991681 PMC2238936

[B15] Guzman E. Taylor G. (2015). Immunology of bovine respiratory syncytial virus in calves. *Mol. Immunol.* 66 48–56. 10.1016/j.molimm.2014.12.004 25553595

[B16] Husen T. de Vlieg J. Alkema W. (2008). BioVenn - a web application for the comparison and visualization of biological lists using area-proportional Venn diagrams. *BMC Genom.* 9:488. 10.1186/1471-2164-9-488 18925949 PMC2584113

[B17] Inchley C. S. Sonerud T. Fjærli H. O. Nakstad B. (2015). Nasal mucosal microRNA expression in children with respiratory syncytial virus infection. *BMC Infect. Dis.* 15:150. 10.1186/s12879-015-0878-z 25884957 PMC4387708

[B18] Inui M. Martello G. S. (2010). MicroRNA control of signal transduction. *Nat. Rev. Mol. Cell Biol.* 11 252–263. 10.1038/nrm2868 20216554

[B19] Janssen R. Pennings J. Hodemaekers H. Buisman A. van Oosten M. de Rond L. (2007). Host transcription profiles upon primary respiratory Syncytial Virus infection. *J. Virol.* 81 5958–5967. 10.1128/jvi.02220-06 17376894 PMC1900269

[B20] Johnston D. Earley B. Cormican P. Murray G. Kenny D. A. Waters S. M. (2017). Illumina MiSeq 16S amplicon sequence analysis of bovine respiratory disease associated bacteria in lung and mediastinal lymph node tissue. *BMC Vet. Res.* 13:118. 10.1186/s12917-017-1035-2 28464950 PMC5414144

[B21] Johnston D. Earley B. McCabe M. S. Lemon K. Duffy C. McMenamy M. (2019). Experimental challenge with bovine respiratory syncytial virus in dairy calves: bronchial lymph node transcriptome response. *Sci. Rep.* 9:14736. 10.1038/s41598-019-51094-z 31611566 PMC6791843

[B22] Johnston D. Kenny D. A. McGee M. Waters S. M. Kelly A. K. Earley B. (2016). Electronic feeding behavioural data as indicators of health status in dairy calves. *Irish J. Agric. Food Res.* 55 159–168. 10.1515/ijafr-2016-0016

[B23] Krämer A. Green J. Pollard J. Tugendreich S. (2014). Causal analysis approaches in ingenuity pathway analysis. *Bioinformatics* 30 523–530. 10.1093/bioinformatics/btt703 24336805 PMC3928520

[B24] Lai Y.-C. Lai Y.-T. Rahman M. M. Chen H.-W. Husna A. A. Fujikawa T. (2020). Bovine milk transcriptome analysis reveals microRNAs and RNU2 involved in mastitis. *FEBS J.* 287 1899–1918. 10.1111/febs.15114 31663680

[B25] Langmead B. Trapnell C. Pop M. Salzberg S. (2009). Ultrafast and memory-efficient alignment of short DNA sequences to the human genome. *Genome Biol.* 10:R25. 19261174 10.1186/gb-2009-10-3-r25 PMC2690996

[B26] Lawless N. Vegh P. O’Farrelly C. Lynn D. J. (2014). The role of microRNAs in bovine infection and immunity. *Front. Immunol.* 5:611. 10.3389/fimmu.2014.00611 25505900 PMC4245999

[B27] Leon-Icaza S. A. Zeng M. Rosas-Taraco A. G. (2019). microRNAs in viral acute respiratory infections: immune regulation, biomarkers, therapy, and vaccines. *ExRNA* 1:1. 10.1186/s41544-018-0004-7 PMC7149109 34171007

[B28] Li Q. Yang C. Du J. Zhang B. He Y. Hu Q. (2018). Characterization of miRNA profiles in the mammary tissue of dairy cattle in response to heat stress. *BMC Genom.* 19:975. 10.1186/s12864-018-5298-1 30593264 PMC6309072

[B29] Luoreng Z.-M. Wang X.-P. Mei C.-G. Zan L.-S. (2018). Comparison of microRNA profiles between bovine mammary glands infected with *Staphylococcus aureus* and *Escherichia coli*. *Intern. J. Biol. Sci.* 14 87–99. 10.7150/ijbs.22498 29483828 PMC5821052

[B30] Martin M. (2011). Cutadapt removes adapter sequences from high-throughput sequencing reads. *EMBnet. J.* 17:10. 10.14806/ej.17.1.200

[B31] Moore E. C. Barber J. Tripp R. A. (2008). Respiratory syncytial virus (RSV) attachment and nonstructural proteins modify the type I interferon response associated with suppressor of cytokine signaling (SOCS) proteins and IFN-stimulated gene-15 (ISG15). *Virol. J.* 5:116. 10.1186/1743-422X-5-116 18851747 PMC2577635

[B32] Murray G. M. More S. J. Sammin D. Casey M. J. McElroy M. C. O’Neill R. G. (2017). Pathogens, patterns of pneumonia, and epidemiologic risk factors associated with respiratory disease in recently weaned cattle in Ireland. *J. Vet. Diagn. Invest.* 29 20–34. 10.1177/1040638716674757 28074713

[B33] Neibergs H. L. Seabury C. M. Wojtowicz A. J. Wang Z. Scraggs E. Kiser J. N. (2014). Susceptibility loci revealed for bovine respiratory disease complex in pre-weaned holstein calves. *BMC Genom.* 15:1164. 10.1186/1471-2164-15-1164 25534905 PMC4445561

[B34] O’Connell R. M. Rao D. S. Chaudhuri A. A. David B. (2010). Physiological and pathological roles for microRNAs in the immune system. *Nat. Rev. Immunol.* 10 111–122. 10.1038/nri2708 20098459

[B35] Othumpangat S. Walton C. Piedimonte G. (2012). MicroRNA-221 modulates RSV replication in human bronchial epithelium by targeting NGF expression. *PLoS One* 7:e30030. 10.1371/journal.pone.0030030 22272270 PMC3260191

[B36] Putz E. J. Putz A. M. Jeon H. Lippolis J. D. Ma H. Reinhardt T. A. (2019). MicroRNA profiles of dry secretions through the first three weeks of the dry period from Holstein cows. *Sci. Rep.* 9:19658. 10.1038/s41598-019-56193-5 31873189 PMC6928067

[B37] Ramaswamy M. Shi L. Monick M. M. Hunninghake G. W. Look D. C. (2004). Specific inhibition of Type I interferon signal transduction by respiratory Syncytial Virus. *Am. J. Respir. Cell Mol. Biol.* 30 893–900. 10.1165/rcmb.2003-0410OC 14722224

[B38] Rima B. Collins P. Easton A. Fouchier R. Kurath G. Lamb R. A. (2017). ICTV Virus taxonomy profile: pneumoviridae. *J. Gen. Virol.* 98 2912–2913. 10.1099/jgv.0.000959 29087278 PMC5775899

[B39] Robinson M. Oshlack A. (2010). A scaling normalization method for differential expression analysis of RNA-seq data. *Genome Biol.* 11:R25. 20196867 10.1186/gb-2010-11-3-r25 PMC2864565

[B40] Robinson M. D. McCarthy D. J. Smyth G. K. (2010). edgeR: a Bioconductor package for differential expression analysis of digital gene expression data. *Bioinformatics* 26 139–140. 10.1093/bioinformatics/btp616 19910308 PMC2796818

[B41] Scheltema N. M. Gentile A. Lucion F. Nokes D. J. Munywoki P. K. Madhi S. A. (2017). Global respiratory syncytial virus-associated mortality in young children (RSV GOLD): a retrospective case series. *Lancet Glob. Health* 5:e00984–91. 10.1016/S2214-109X(17)30344-3 PMC5599304 28911764

[B42] Solberg O. D. Ostrin E. J. Love M. I. Peng J. C. Bhakta N. R. Hou L. (2012). Airway epithelial miRNA expression is altered in asthma. *Am. J. Respir. Crit. Care Med.* 186 965–974. 10.1164/rccm.201201-0027OC 22955319 PMC3530212

[B43] Spann K. M. Tran K. C. Collins P. L. (2005). Effects of nonstructural proteins NS1 and NS2 of human respiratory syncytial virus on interferon regulatory factor 3, NF-kappaB, and proinflammatory cytokines. *J. Virol.* 79 5353–5362. 10.1128/JVI.79.9.5353-5362.2005 15827150 PMC1082743

[B44] Sudaryatma P. E. Nakamura K. Mekata H. Sekiguchi S. Kubo M. Kobayashi I. (2018). Bovine respiratory syncytial virus infection enhances *Pasteurella multocida* adherence on respiratory epithelial cells. *Vet. Microbiol.* 220 33–38. 10.1016/j.vetmic.2018.04.031 29885798 PMC7117154

[B45] Taxis T. M. Bauermann F. V. Ridpath J. F. Casas E. (2017). Circulating MicroRNAs in serum from cattle challenged with bovine viral diarrhea Virus‡. *Front. Genet.* 8:91. 10.3389/fgene.2017.00091 28702050 PMC5487392

[B46] Taylor J. D. Fulton R. W. Lehenbauer T. W. Step D. L. Confer A. W. (2010). The epidemiology of bovine respiratory disease: what is the evidence for predisposing factors? *Can. Vet. J.* 51 1095–1102. 21197200 PMC2942046

[B47] Thornburg N. J. Hayward S. L. Crowe J. E. Jr. (2012). Respiratory syncytial virus regulates human microRNAs by using mechanisms involving beta interferon and NF-κB. *mBio* 3:e00220–12. 10.1128/mBio.00220-12 23249809 PMC3529541

[B48] Tizioto P. C. Kim J. Seabury C. M. Schnabel R. D. Gershwin L. J. Van Eenennaam A. L. (2015). Immunological response to single pathogen challenge with agents of the bovine respiratory disease complex: an RNA-sequence analysis of the bronchial lymph node transcriptome. *PLoS One* 10:e0131459. 10.1371/journal.pone.0131459 26121276 PMC4484807

[B49] Valarcher J.-F. Taylor G. (2007). Bovine respiratory syncytial virus infection. *Vet. Res.* 38 153–180. 17257568 10.1051/vetres:2006053

[B50] Vegh P. Foroushani A. B. K. Magee D. A. McCabe M. S. Browne J. A. Nalpas N. C. (2013). Profiling microRNA expression in bovine alveolar macrophages using RNA-seq. *Vet. Immunol. Immunopathol.* 155 238–244. 10.1016/j.vetimm.2013.08.004 24021155

[B51] Vieira S. E. Bando S. Y. de Paulis M. Oliveira D. B. L. Thomazelli L. M. Durigon E. L. (2019). Distinct transcriptional modules in the peripheral blood mononuclear cells response to human respiratory syncytial virus or to human rhinovirus in hospitalized infants with bronchiolitis. *PLoS One* 14:e0213501. 10.1371/journal.pone.0213501 30845274 PMC6405118

[B52] Wahid F. Shehzad A. Khan T. Kim Y. Y. (2010). MicroRNAs: synthesis, mechanism, function, and recent clinical trials. *Biochim. Biophys. Acta Mol. Cell Res.* 1803 1231–1243. 10.1016/j.bbamcr.2010.06.013 20619301

[B53] Wu W. Choi E.-J. Lee I. Lee Y. S. Bao X. (2020). Non-Coding RNAs and their role in Respiratory Syncytial Virus (RSV) and Human Metapneumovirus (hMPV) infections. *Viruses* 12:345. 10.3390/v12030345 32245206 PMC7150941

[B54] Zhang B. Chen L. Silacci C. Thom M. Boyington J. C. Druz A. (2017). Protection of calves by a prefusion-stabilized bovine RSV F vaccine. *NPJ Vac.* 2:7. 10.1038/s41541-017-0005-9 29021918 PMC5627276

[B55] Zheng J. Yang P. Tang Y. Pan Z. Zhao D. (2015). Respiratory Syncytial Virus nonstructural proteins upregulate SOCS1 and SOCS3 in the different manner from endogenous IFN signaling. *J. Immunol. Res.* 2015:738547. 10.1155/2015/738547 26557722 PMC4628668

